# The POLD1^R689W^ variant increases the sensitivity of colorectal cancer cells to ATR and CHK1 inhibitors

**DOI:** 10.1038/s41598-020-76033-1

**Published:** 2020-11-03

**Authors:** Albert Job, Marina Tatura, Cora Schäfer, Veronika Lutz, Hanna Schneider, Brigitte Lankat-Buttgereit, Alexandra Zielinski, Kerstin Borgmann, Christian Bauer, Thomas M. Gress, Malte Buchholz, Eike Gallmeier

**Affiliations:** 1 Department of Gastroenterology, Endocrinology, Metabolism, and Infectiology, University Hospital of Marburg, Philipps-University Marburg, Baldingerstraße, 35043 Marburg, Germany; 2 Lab of Radiobiology & Experimental Radiooncology, University Medical Center Hamburg-Eppendorf, Hamburg, Germany

**Keywords:** Cancer, Molecular biology, Biomarkers, Diseases, Medical research, Molecular medicine, Oncology

## Abstract

Inhibition of the kinase ATR, a central regulator of the DNA damage response, eliminates subsets of cancer cells in certain tumors. As previously shown, this is at least partly attributable to synthetic lethal interactions between *ATR* and *POLD1*, the catalytic subunit of the polymerase δ. Various *POLD1* variants have been found in colorectal cancer, but their significance as therapeutic targets for ATR pathway inhibition remains unknown. Using CRISPR/Cas9 in the colorectal cancer cell line DLD-1, which harbors four *POLD1* variants, we established heterozygous *POLD1*-knockout clones with exclusive expression of distinct variants to determine the functional relevance of these variants individually by assessing their impact on ATR pathway activation, DNA replication, and cellular sensitivity to inhibition of ATR or its effector kinase CHK1. Of the four variants analyzed, only POLD1^R689W^ affected POLD1 function, as demonstrated by compensatory ATR pathway activation and impaired DNA replication. Upon treatment with ATR or CHK1 inhibitors, POLD1^R689W^ strongly decreased cell survival in vitro, which was attributable at least partly to S phase impairment and apoptosis. Similarly, treatment with the ATR inhibitor AZD6738 inhibited growth of murine xenograft tumors, harboring the POLD1^R689W^ variant, in vivo. Our *POLD1*-knockout model thus complements algorithm-based models to predict the pathogenicity of tumor-specific variants of unknown significance and illustrates a novel and potentially clinically relevant therapeutic approach using ATR/CHK1 inhibitors in *POLD1*-deficient tumors.

## Introduction

The relationship between two genes, in which single mutations alone are not lethal, but in combination are incompatible with cell survival, is defined as synthetic lethality^1,2^. As tumors frequently harbor defects in genes involved in DNA repair^3^ and those defects are often compensated by other DNA repair genes^3,4^, synthetic lethality represents a novel approach for the individualized genotype-based treatment of tumors by pharmacologically targeting the compensatory partner of defective DNA repair genes. One prominent example of such an approach is the treatment of patients harboring *BRCA1/2*-deficient cancers with PARP inhibitors^5^.

ATR is a phosphoinositide 3-kinase-related kinase and acts as central regulator of the replication checkpoint during DNA damage response^6^. Activated by the accumulation of single-stranded DNA at sites of replication stress or DNA damage, ATR initiates replication fork stabilization, cell cycle arrest and DNA repair via homologous recombination (HR)^6,7^. Inhibitors of ATR are currently investigated in clinical trials^8,9^ and appear to enable the effective elimination of certain subsets of cancer cells. Despite the identification of a variety of potential biomarkers (comprehensively reviewed by Lecona & Fernandez-Capetillo^7^), the specific determinants of this therapeutic response remain incompletely defined, particularly in regard to synthetically lethal relationships between *ATR* and certain DNA repair genes.

We and others previously identified *POLD1* as acting synthetically lethal with *ATR*^10,11^. POLD1 is the catalytic subunit of the polymerase (Pol) δ which is responsible for the elongation of the lagging strand during DNA replication. Related essential components of DNA replication are the Polα-primase complex and Polε^12,13^. *POLD1* variants have been recently identified in colorectal and other cancers^14–16^. The significance of these variants as potential biomarkers for the targeted treatment of tumors with ATR inhibitors, however, remains enigmatic.

Therefore, we applied CRISPR/Cas9 to establish a *POLD1*-knockout (KO) model in the human colorectal cancer (CRC) cell line DLD-1, which harbors four heterozygous *POLD1* variants^17^, yielding cell clones with exclusive expression of distinct *POLD1* variants. On the one hand, this model facilitated the determination of the functional impact of specific *POLD1* variants and could thus functionally complement existing prediction models for the pathogenicity of variants of unknown significance (VUS). On the other hand, it allowed defining the impact of different *POLD1* variants on the sensitivity to inhibitors of ATR or other components of the ATR pathway in vitro and in vivo.

## Results

### CRISPR/Cas9-mediated generation of a *POLD1*-knockout model

**Figure 1:**
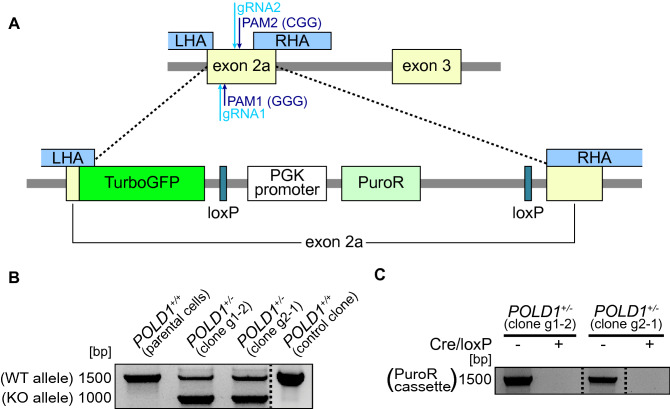
CRISPR/Cas9-mediated generation of a *POLD1*-knockout model. **(A)** Schematic overview of the targeting procedure, displaying CRISPR/Cas9-mediated integration of the repair template in exon 2a of the *POLD1* locus. **(B)** PCR detecting the *POLD1*-KO and –WT alleles as well as **(C)** the Cre/loxP-mediated excision of the puromycin resistance cassette. Dashed lines indicate cropping, and the original gels are displayed in Figure S1.

To establish a *POLD1*-KO in the DLD-1 cell line, we used the CRISPS/Cas9 technique to integrate a repair template into exon 2a of the *POLD1* locus via HR (Fig. 1A), causing allele inactivation. Multiple single-cell colonies were genotyped and two distinct heterozygous *POLD1*-KO clones obtained (termed g1-2 and g2-1). Clones derived from scrambled guide RNA (gRNA)-transfected cells served as *POLD1*^+*/*+^ control (ctrl) (Fig. 1B). Immunoblotting of g1-2 and g2-1 revealed no changes in POLD1 protein expression as compared to *POLD1*^+*/*+^ parental cells and the ctrl clone (data not shown). To enable the consecutive generation of homozygous *POLD1*-KO clones, we excised the floxed puromycin resistance cassette (Fig. 1A) in both g1-2 and g2-1 cell clones using Cre recombinase (Fig. 1C). Upon targeting of the second allele, however, no homozygous *POLD1*-KO clones were obtained despite multiple targeting rounds.

### Identification of the allele-specific localization of previously reported *POLD1* variants in DLD-1

**Figure 2:**
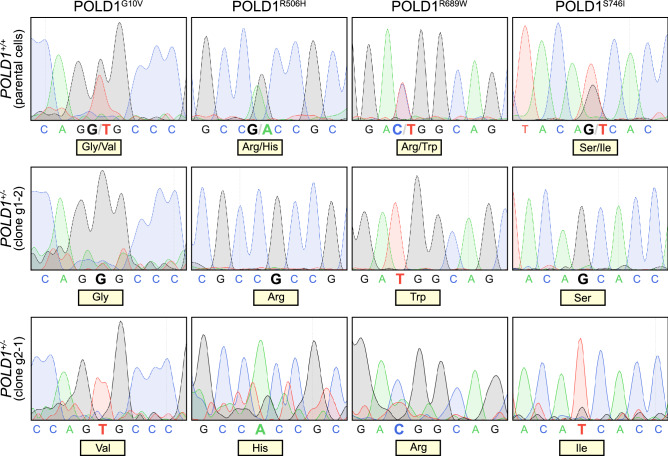
Identification of the allele-specific localization of previously reported *POLD1* variants in DLD-1. Sequencing of *POLD1* variants in *POLD1*^+*/*+^ parental cells (upper panel) as well as the *POLD1*^+*/-*^ g1-2 (middle panel) and g2-1 (lower panel) cell clones.

Four heterozygous *POLD1* variants have been reported in DLD-1 cells, namely G10V, R506H, R689W and S746I^17^. These were confirmed in our DLD-1 *POLD1*^+*/*+^ cells by genome sequencing (Fig. 2, upper panel). However, the allele specific localization of these variants remained unknown. Using mRNA-sequencing, we demonstrated that the intact *POLD1* allele of the heterozygous cell clone g1-2 exhibits only the R689W variant (termed *POLD1*^*R689W/-*^, Fig. 2, middle panel), while the intact *POLD1* allele of the heterozygous cell clone g2-1 exhibits the other three variants G10V, R506H and S746I (*POLD1*^*G10V,R506H,S746I/-*^, according to the data obtained later termed here *POLD1*^+*/-*^ for convenience, Fig. 2, lower panel).

### Functional characterization of the pre-existing *POLD1* variants in DLD-1

**Figure 3:**
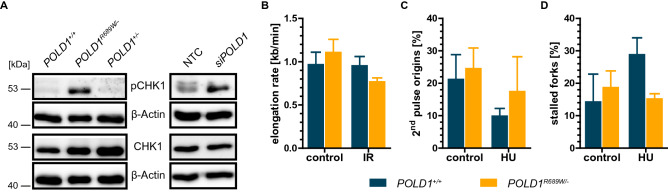
Functional characterization of the pre-existing *POLD1* variants in DLD-1.** (A)** Representative results of constitutive protein levels (n = 3) in *POLD1*^+*/*+^, *POLD1*^*R689W/-*^ and *POLD1*^+*/-*^ cells as well as protein levels 120 h after *siPOLD1* transfection in *POLD1*^+*/*+^ cells by immunoblotting. β-Actin served as loading control and original immunoblots are displayed in Figure S2. **(B)** Quantification of the elongation rate, **(C)** 2nd pulse origins and **(D)** stalled replication forks upon treatment of *POLD1*^+*/*+^ and *POLD1*^*R689W/-*^ cells with IR or HU via DNA fiber assay. Error bars represent mean ± SEM of three independent experiments with at least 100 DNA fibers analyzed in each experiment. Using a two-tailed, unpaired Student’s *t* test, statistical significance could not be reached.

As defects in DNA replication lead to the activation of DNA repair pathways^4^, the functional inactivation of POLD1 is expected to cause compensatory activation of the ATR pathway^10^. We therefore assessed protein and phosphorylation levels of CHK1, the major effector kinase of ATR, to determine the functional significance of the pre-existing *POLD1* variants in DLD-1. We observed a strong constitutive CHK1 phosphorylation exclusively in *POLD1*^*R689W/-*^ but not in *POLD1*^+*/-*^ and *POLD1*^+*/*+^ cells (Fig. 3A, left panel), an effect qualitatively comparable to the effects of small interfering RNA (siRNA)-mediated subtotal *POLD1*-depletion (Fig. 3A, right panel). Therefore, only the POLD1^R689W^ variant had a measurable impact on POLD1 function in regard to compensatory ATR/CHK1 pathway activation, while the other variants POLD1^G10V^, POLD1^R506H^ and POLD1^S746I^ caused no discernible effect. As *POLD1* encodes the catalytic subunit of Polδ, we next investigated the impact of this POLD1^R689W^ variant on DNA replication upon treatment with ionizing radiation (IR) or hydroxyurea (HU), using DNA fiber assays. We found that the constitutive overall elongation rate was slightly higher in *POLD1*^*R689W/-*^ than in *POLD1*^+*/*+^ cells. Upon IR-treatment, however, the elongation rate decreased in *POLD1*^*R689W/-*^ cells, but remained unchanged in *POLD1*^+*/*+^ cells (Fig. 3B). Upon HU-treatment, 2^nd^ pulse origins were decreased to a higher extent in *POLD1*^+*/*+^ than in *POLD1*^*R689W/-*^ cells (Fig. 3C). Consistently, stalled replication forks were increased only in *POLD1*^+*/*+^ but not in *POLD1*^*R689W/-*^ cells (Fig. 3D). Although, these data did not reach statistical significance, there appeared to be a clear trend suggesting that the POLD1^R689W^ variant functionally impaired the stability of DNA fibers, as shown by the decreased elongation rate upon IR, while having no effect on the ongoing replication process despite replicational stress, as demonstrated by virtually unchanged second pulse origins and stalled replication forks specifically in *POLD1*^*R689W/-*^ cells.

### Mechanistic characterization of different *POLD1* variants in DLD-1

**Figure 4:**
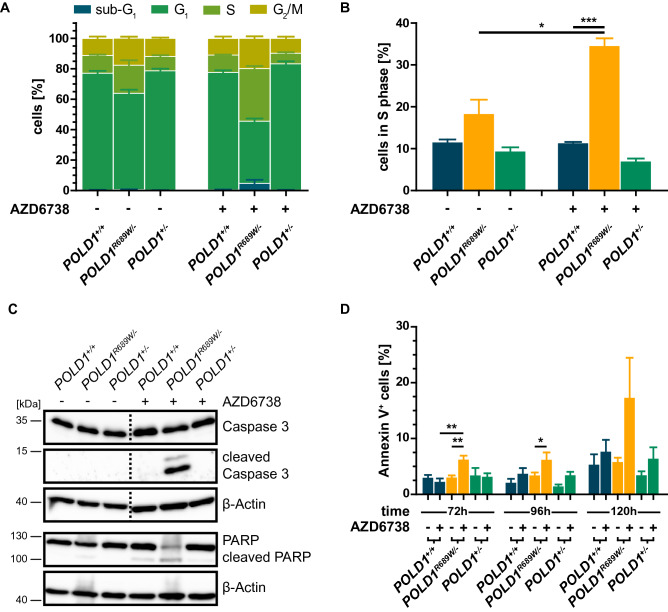
Mechanistic characterization of the pre-existing *POLD1* variants in DLD-1. **(A)** Cell cycle profile (n = 3) of DLD-1 *POLD1*^+*/*+^, *POLD1*^*R689W/-*^, and *POLD1*^+*/-*^ cells 96 h after treatment with 1 µM AZD6738 as assessed by flow cytometry. **(B)** S phase fraction of the cell cycle profile from **(A)** in detail. **(C)** Representative results of protein levels (n = 5) in *POLD1*^+*/*+^, *POLD1*^*R689W/-*^, and *POLD1*^+*/-*^ cells by immunoblotting 72 h after treatment with 1 µM AZD6738. β-Actin served as loading control. Dashed lines indicate cropping, and the original immunoblots are displayed in Figure S3. **(D)** Quantification (n = 5) of annexin V^+^ apoptotic cells (including early apoptotic PI^-^/annexin V^+^ cells and late apoptotic PI^+^/annexin V^+^ cells) in *POLD1*^+*/*+^, *POLD1*^*R689W/-*^, and *POLD1*^+*/-*^ cells at 72 h, 96 h and 120 h after treatment with 1 µM AZD6738 as assessed by flow cytometry. Statistically significant outliers were identified by Grubbs’ test and excluded. Error bars represent mean ± SEM, and asterisks (* p < 0.05, ** p < 0.01, *** p < 0.001) mark statistical significance using a one-tailed **(D)** or two-tailed **(B)**, unpaired Student’s *t* test.

To analyze the molecular mechanism underlying R689W-mediated impairment of POLD1 function, we compared the cell cycle profiles of *POLD1*^+*/*+^, *POLD1*^*R689W/-*^ and *POLD1*^+*/-*^ cells upon treatment with the ATR inhibitor AZD6738 (Fig. 4A). Constitutively, *POLD1*^*R689W/-*^ cells displayed a slight increase of the S phase fraction compared to *POLD1*^+*/*+^ and *POLD1*^+*/-*^ cells (Fig. 4A + B). Treatment with the ATR inhibitor AZD6738 resulted in an additional, significant increase of the S phase fraction exclusively in *POLD1*^*R689W/-*^ cells. Furthermore, we found an increase of the sub-G_1_ fraction exclusively in *POLD1*^*R689W/-*^ cells upon AZD6738 treatment, indicating that not only cell cycle perturbations but also apoptosis appeared to contribute to the POLD1^R689W^-dependent effects of ATR inhibition on cell viability. Consistently, we observed cleavage of caspase 3, the main effector protease of apoptosis, and its substrate PARP nearly exclusively in *POLD1*^*R689W/-*^ cells upon treatment with AZD6738 (Fig. 4C). To quantify the extent of apoptosis, we next applied annexin V-staining at 72 h, 96 h and 120 h after treatment with AZD6738 in *POLD1*^+*/*+^, *POLD1*^*R689W/-*^ and *POLD1*^+*/-*^ cells. We observed a significant increase of annexin V^+^ cells in AZD6738-treated *POLD1*^*R689W/-*^ 72 h and 96 h post-treatment as compared to AZD6738-treated *POLD1*^+*/*+^ or untreated *POLD1*^*R689W/-*^ cells, respectively, an effect which numerically increased strongly further (approximately threefold) but did not reach statistical significance at 120 h post-treatment (Fig. 4D). Thus, the detrimental effects of POLD1^R689W^ observed upon treatment with the ATR inhibitor AZD6738 are mechanistically at least partially attributable to an S phase impairment and apoptosis.

### POLD1^R689W^-mediated sensitization of DLD-1 cells to ATR and CHK1 inhibitors in vitro

**Figure 5:**
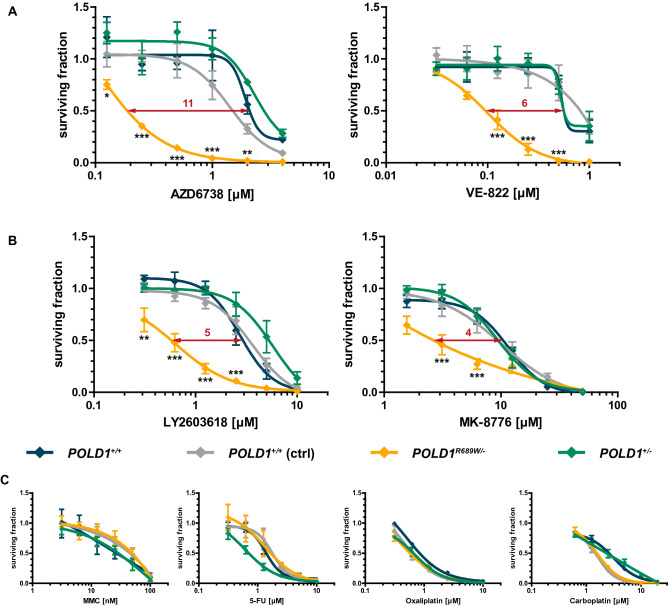
POLD1^R689W^-mediated sensitization of DLD-1 cells to ATR and CHK1 inhibitors in vitro. **(A)** Effects of ATR inhibitors (n = 3), **(B)** CHK1 inhibitors (n = 4), and **(C)** common chemotherapeutics (n = 3 for MMC and carboplatin; n = 4 for 5-FU and oxaliplatin) on the proliferation of DLD-1 *POLD1*^*R689W/-*^ cells as compared to *POLD1*^+*/-*^ and *POLD1*^+*/*+^ parental and ctrl cells, measured 120 h after drug treatment. Data are presented as mean ± SEM and each data point reflects triplicate wells of multiple independently performed experiments. Asterisks (* p < 0.05, ** p < 0.01, *** p < 0.001) mark statistical significance using a two-way ANOVA with Bonferroni post-test.

Having established a functional role of the POLD1^R689W^ variant and its mechanistic impact on treatment with the ATR inhibitor AZD6738, we next tested, whether these effects were generalizable to other inhibitors targeting either ATR or its main effector kinase CHK1. Compared to parental and ctrl *POLD1*^+*/*+^ cells, only *POLD1*^*R689W/-*^ but not *POLD1*^+*/-*^ cells displayed an increased sensitivity not only to AZD6738, but also to another ATR inhibitor VE-822^8,9^ with IC_50_ ratios of 11 and 6, respectively (Fig. 5A). Similarly, only *POLD1*^*R689W/-*^ but not *POLD1*^+*/-*^ cells displayed an increased sensitivity to the CHK1 inhibitors LY2603618 and MK-8776^18–20^ with IC_50_ ratios of 5 and 4, respectively (Fig. 5B). To exclude an unspecific drug hypersensitivity phenotype, the cells were additionally treated with common chemotherapeutics including mitomycin C (MMC), 5-fluorouracil (5-FU), carboplatin and oxaliplatin, none of which caused POLD1^R689W^-dependent inhibition of proliferation (Fig. 5C). Thus, only POLD1^R689W^ but not POLD1^G10V^, POLD1^R506H^ or POLD1^S746I^ sensitizes DLD-1 cells specifically to ATR and CHK1 inhibitors, but not to common chemotherapeutics.

### POLD1^R689W^-mediated sensitization of DLD-1 cells to the ATR inhibitor AZD6738 in vivo

**Figure 6:**
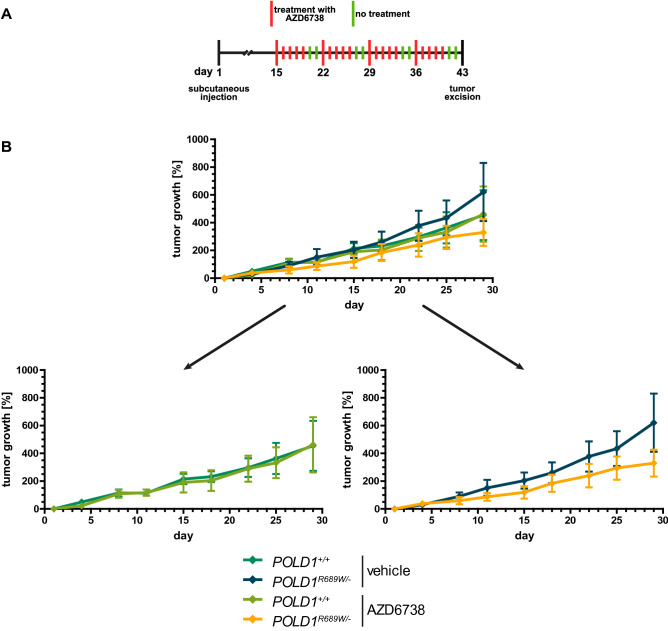
POLD1^R689W^-mediated sensitization of DLD-1 cells to the ATR inhibitor AZD6738 in vivo. **(A)** Schematic representation of the experimental procedure. For clarity, the datasets illustrating vehicle- and AZD6738-treated *POLD1*^+*/*+^ tumors **(B**, lower left panel**)** as well as vehicle- and AZD6738-treated *POLD1*^*R689W/-*^ tumors **(B**, lower right panel**)** are displayed separately. Error bars represent mean ± SEM of eight (vehicle-treated *POLD1*^*R689W/-*^ tumors) or nine mice (vehicle-treated *POLD1*^+*/*+^ tumors, AZD6738-treated *POLD1*^+*/*+^ tumors, and AZD6738-treated *POLD1*^*R689W/-*^ tumors), respectively.

To extend our in vitro data we next used a murine xenograft tumor model to assess the sensitivity of *POLD1*^+*/*+^ and *POLD1*^*R689W/-*^ cells to the ATR inhibitor AZD6738 in vivo, as this inhibitor showed the strongest proliferation inhibitory effects in the in vitro experiments (Fig. 5A + B). Female nude mice were treated two weeks after subcutaneous injection of DLD-1 *POLD1*^+*/*+^ or *POLD1*^*R689W/-*^ cells with the ATR inhibitor AZD6738 or vehicle once daily for five consecutive days, followed by two days without treatment, over a time period of four weeks (Fig. 6A). When comparing vehicle- and AZD6738-treated *POLD1*^+*/*+^ tumors, we observed virtually no discernible differences in tumor growth, with both quintupling their original tumor size during treatment (Fig. 6B, upper and lower left panel). In contrast, while the overall strongest tumor growth (> 600%) was observed in vehicle-treated *POLD1*^*R689W/-*^ tumors, AZD6738-treated *POLD1*^*R689W/-*^ tumors showed the slowest tumor growth (Fig. 6B, upper and lower right panel), although these data did not reach statistical significance. Nevertheless, these data at least support the hypothesis that AZD6738 decreases tumor growth specifically of *POLD1*^*R689W/-*^ tumors in vivo. Taken together with our in vitro data, these results illustrate the general suitability of a cellular *POLD1*-KO model system to define *POLD1* VUS as either pathogenic or non-pathogenic, as has similarly been shown for *BRCA2* VUS^21^.

## Discussion

Synthetic lethality offers new approaches for a targeted and individualized tumor therapy with reduced side effects^1,2^. By screening an siRNA library, we previously identified *POLD1*, coding for the catalytic subunit of Polδ, to act synthetically lethal with *ATR*, a DNA damage sensing kinase^10^. Recently, variants in *POLD1* have been identified in colorectal and other cancers^14–16^. Therefore, we established a *POLD1*-KO model system to determine the pathogenicity of individual *POLD1* variants and their impact on the sensitivity to inhibitors of the ATR pathway in vitro and in vivo.

DLD-1, a human CRC cell line, harbors the four heterozygous *POLD1* variants G10V, R506H, R689W and S746I^17^. We applied the CRISPR/Cas9 technique in order to separately disrupt each *POLD1* allele followed by genome/mRNA sequencing to identify the allele-specific localization of the above variants, yielding KO clones expressing either POLD1^G10V/R506H/S746I^ (termed *POLD1*^+*/-*^) or POLD1^R689W^ (termed *POLD1*^*R689W/-*^), respectively. This approach provides a well-suited model system for the functional characterization of *POLD1* VUS. Despite multiple targeting rounds, we were not able to establish a homozygous *POLD1*^*-/-*^ clone, indicating *POLD1* to be an essential gene in cancer cells. This hypothesis is further supported by a study of Uchimura et al.^22^, demonstrating in a murine KO model that *POLD1* is essential during embryonic development.

In our system, only POLD1^R689W^ but not POLD1^G10V/R506H/S746I^ displayed functional significance with regard to compensatory activation of the ATR pathway, as shown by strongly increased CHK1 phosphorylation as a surrogate marker for DNA replication stress, exclusively in *POLD1*^*R689W/-*^ cells. Transferred to a clinical setting, CHK1 phosphorylation could thus provide a predictive biomarker for increased sensitivity to ATR inhibitors^7^. Consistently, the *S. cerevisiae* analog of POLD1^R689W^ (POLD1^R696W^) causes catastrophic genomic instability and a mutator phenotype in yeast^23–25^.

To assess the impact of POLD1^R689W^ during DNA replication more accurately, we also applied DNA fiber assays. We observed a constitutively increased elongation rate in *POLD1*^*R689W/-*^ cells, which could be attributable to a reduction of nucleotide selectivity caused by POLD1^R689W^ and its mediated increase of nucleotide levels as has been demonstrated previously in yeast and also other human cancer cell lines^23–25^. In contrast, IR-treatment led to a decreased elongation rate in *POLD1*^*R689W/-*^ cells. This could be explained by an increased mutational burden caused by additional induction of extrinsic DNA damage in cells that already harbor a mutator phenotype – predominantly GC→TA transversions and GC→AT transitions—due to POLD1^R689W^^24,25^, ultimately leading to irreversible instability of DNA fibers. Unexpectedly, POLD1^R689W^ did not have an impact on the ongoing replication process, even upon treatment with HU, which inhibits the ribonucleotide reductase^26^ resulting in decreased levels of nucleotides and eventually a blocked replication process. This could be explained by the previously described POLD1^R689W^-induced increase of nucleotide levels^24^, consecutively antagonizing the effects of HU.

Investigating the role of cell cycle impairment or apoptosis as potential molecular mechanisms for the increased sensitivity of *POLD1*^*R689W/-*^ cells to ATR/CHK1 inhibitors, we observed a constitutive slight increase of the S phase fraction in *POLD1*^*R689W/-*^ cells, which significantly increased upon AZD6738 treatment. Perhaps even more importantly, AZD6738-treatment also induced apoptosis in *POLD1*^*R689W/-*^ cells as indicated by the increased sub-G_1_ fraction in the cell cycle experiments, and consecutively verified by detection of cleaved caspase 3 and its substrate PARP, as well as by quantification of annexin V^+^ apoptotic cells. Interestingly, the observed S phase impairment along with caspase 3-mediated apoptosis in *POLD1*-deficient colorectal cancer cells upon AZD6738 treatment has similarly been demonstrated in *ATM*-dysfunctional gastric cancer cells upon AZD6738 treatment, supporting the known synthetically lethal relationship between *ATR* and *ATM*^27^.

We next assessed the impact of POLD1^R689W^ on the cellular sensitivity to ATR pathway-inhibiting chemical agents in vitro, using clinically relevant inhibitors of ATR^8,9^ and CHK1^18–20^. In fact, both ATR and CHK1 inhibitors decreased proliferation specifically of *POLD1*^*R689W/-*^ but not *POLD1*^+*/-*^ cells. Importantly, no significant proliferation differences were observable between these cells upon treatment with common chemotherapeutics, excluding a general drug hypersensitivity phenotype of *POLD1*^*R689W/-*^ cells. As an extension to our in vitro data, we further tested the effects of the ATR inhibitor AZD6738 on *POLD1*^*R689W/-*^ cells in a murine xenograft tumor model in vivo. Although the tumor growth rates did not differ in a statistically significant manner overall among the four groups (vehicle- versus AZD6738-treatment of *POLD1*^+*/*+^ versus *POLD1*^*R689W/-*^ tumors), a discernible inhibition of tumor growth upon AZD6738 treatment was only observable in *POLD1*^*R689W/-*^ tumors, while *POLD1*^+*/*+^ tumors displayed virtually identical tumor growth rates when compared to vehicle-treated animals. While statistically, these results do not allow conclusions beyond the recognition of a trend, the reasons for the weaker effects of our in vivo as compared to our in vitro data might simply be attributable to typical problems inherent to animal models such as route of administration, dosing or certain artifacts, and merit further studies in modified in vivo models. In addition, future studies should address the impact of other ATR and CHK1 inhibitors in vivo—including VE-822, LY2603618, and MY-8776—to allow a more accurate estimation of the potential clinical relevance of our data, and as a prerequisite prior to the establishment of clinical trials.

In addition, we compared our functional data defining the significance of the investigated *POLD1* variants with predictive data from various pathogenicity tools, including PON-P2, a machine learning-based classified, which groups variants into pathogenic, neutral, or unknown classes, based on random forest probability score^28,29^; PolyPhen-2, calculating the naïve Bayes posterior probability of a variant’s impact on protein function to be possibly/probably damaging or benign (non-damaging)^30,31^; PROVEAN, which uses an alignment-based score to discriminate between deleterious and neutral effects on protein function^32,33^; and MutationAssessor, calculating a functional impact score by using evolutionary conservation patterns^34,35^. According to these prediction tools, POLD1^G10V^ and POLD1^S746I^ variants have no or only low functional impact (Table 1), which is in concordance with our data on these variants. In contrast, POLD1^R506H^ and POLD1^R689W^ are both predicted by PON-P2 and PROVEAN to be pathogenic, while they are differently classified by PolyPhen-2 and MutationAssessor regarding their damaging potential and functional impact score, respectively, with POLD1^R689W^ achieving a stronger functional impairment. However, despite the predicted functional significance and pathogenicity of POLD1^R506H^, we demonstrated that in our model only POLD1^R689W^ had a functional impact in regard to CHK1 phosphorylation, DNA replication, cell cycle impairment, apoptosis induction, and cellular sensitivity to inhibitors of the ATR pathway. Besides the obvious interpretation, i.e. the false classification of these VUS by either the prediction tools or our assays, these inconsistencies could also be explained by the fact that we did not analyze the POLD1^R506H^ variant alone, but only in combination with the variants POLD1^G10V^ and POLD1^S746I^, which could cause antagonizing or protective effects in terms of “revertant” variants, a phenomenon previously described for *BRCA2* in a similar clinical context^36,37^. This hypothesis should be tested in future studies through the individual introduction of single *POLD1* variants (including the *POLD1*^*R506H*^ variant), and consecutive characterization of their functional relevance in colorectal cancer cells. Nevertheless, the combination of algorithm-based, theoretical prediction tools to identify pathogenic variants along with functional testing in suitable KO/Knock-in models likely represents the most accurate approach currently available to estimate pathogenicity and define clinical-therapeutic exploitability of specific VUS in tumors.

**Table 1:** Pathogenicity for *POLD1* variants according to different prediction tools with investigated variants are in bold.

		Prediction tool			
Variant	References	PON-P2	PolyPhen-2	PROVEAN	Mutation assessor
**p.G10V** **c.29G > T**	^17^	**Unknown (0.398)**	**Benign (0.041)**	**Neutral (− 1.407)**	**Neutral (0.55)**
p.V295M c.883G > A	^16^	Unknown (0.703)	Benign (0.410)	Neutral (0.412)	Neutral (0.09)
p.D316H c.946G > C	^16^	Pathogenic (0.941)	Probably damaging (1.000)	Deleterious (− 6.857)	High (3.69)
p.D316G c.947A > G	^16^	Pathogenic (0.919)	Probably damaging (1.000)	Deleterious (− 6.857)	High (3.69)
p.P327L c.981C > G	^14^	Pathogenic (0.947)	Probably damaging (0.999)	Deleterious (− 9.824)	High (3.74)
p.S370R c.1110C > G	^14^	Unknown (0.560)	Benign (0.407)	Deleterious (− 4.259)	Medium (3.115)
p.R409W c.1225C > T	^16^	Pathogenic (0.963)	Probably damaging (1.000)	Deleterious (− 7.859)	High (3.74)
p.G426S c.1276G > A	^14^	Unknown (0.237)	Benign (0.042)	Neutral (− 0.579)	Neutral (− 0.485)
p.L474P c.1421T > C	^15,16^	Pathogenic (0.937)	Probably damaging (1.000)	Deleterious (− 6.735)	High (3.74)
p.S478N c.1433G > A	^14–16^	Pathogenic (0.759)	Probably damaging (0.998)	Deleterious (− 2.820)	High (4.215)
**p.R506H** **c.1517G > A**	^17^	**Pathogenic (0.955)**	**Possibly damaging (0.755)**	**Deleterious (− 4.922)**	**Medium (2.86)**
p.R521Q c.1562G > A	^16^	Pathogenic (0.957)	Possibly damaging (0.816)	Deleterious (− 3.464)	Medium (3.255)
**p.R689W** **c.2065C > T**	^17^	**Pathogenic (0.973)**	**Probably damaging (0.987)**	**Deleterious (− 7.978)**	**High (4.56)**
**p.S746I** **c.2237G > T**	^17^	**Unknown (0.475)**	**Benign (0.143)**	**Deleterious (− 3.178)**	**Medium (2.92)**

Multiple heterozygous germline variants in *POLD1* have been identified in various cancer types, including colon, endometrium, breast, or brain tumors^14–16^. Most of those variants that are predicted to be functionally significant (Table 1) are located in the proofreading exonuclease domain of POLD1, presumably impairing the repair capacity of Polδ, and thereby conferring a mutator phenotype^24^. In contrast, the role of variants in the polymerase active domain of POLD1 is less well understood. In addition to POLD1^R689W^^24,25^ only one further variant within the polymerase active domain, namely POLD1^L604K^ of murine Polδ, has been described to be potentially pathogenic^38^. Yet, there is growing evidence that base selectivity defects in tumors—as induced by POLD1^R689W^—might result in dramatic consequences for genome stability^24^. Our data now add another example that variants not only within the proofreading exonuclease domain but also in the polymerase active domain of POLD1, e.g. R689W, are functionally relevant and could thus confer a pathogenic phenotype.

In summary, we used CRISPR/Cas9 to establish a *POLD1*-KO model for the functional characterization of *POLD1* VUS. This model could complement and improve the accuracy of algorithm-based theoretical prediction models of *POLD1* VUS pathogenicity. In this model, we demonstrated and mechanistically characterized the impact of the POLD1^R689W^ variant on the cellular sensitivity to ATR and CHK1 inhibitors in vitro, confirming the previously described synthetically lethal relationship between *ATR* and *POLD1*^10^*.* In addition, we demonstrated in a murine in vivo model that treatment with the ATR inhibitor AZD6738 caused a discernible inhibition of tumor growth rate specifically in tumors harboring the POLD1^R689W^ variant. Taken together, our study defines the POLD1^R689W^ variant as target for ATR pathway inhibition and thereby illustrates the emerging role of tumor-specific alterations in polymerases^39–43^ along with the need of their comprehensive identification and functional characterization in suitable model systems to aid the development of novel and rational approaches towards improved tumor therapy^44^.

## Methods

### Cell lines and culture conditions

The human CRC cell line DLD-1 was purchased from the Leibniz Institut DSMZ (Braunschweig, Germany). All cell lines and clones were maintained in Roswell Park Memorial Institute (RPMI 1640) medium supplemented with 10% fetal bovine serum (FBS) and incubated at 37 °C and 5% CO_2_.

### Drugs

AZD6738 and VE-822 were purchased from MedKoo Biosciences (Morrisville, NC, USA), MK-8776 and LY2603618 from Selleckchem (Munich, Germany), and MMC and 5-FU from Sigma-Aldrich (Hamburg, Germany). Oxaliplatin and carboplatin were kindly donated from the cytostatic drug department of the University Hospital Marburg. AZD6738, used in the xenograft in vivo assay, was purchased from AdooQ Bioscience (Irvine, CA, USA).

### Transfection

Reverse transfection was used for the transfection experiments. siRNA targeting *POLD1* (CGGGACCAGGGAGAATTAATA) (Qiagen, Hilden, Germany) at a final concentration of 10 nM or 1 μg plasmid DNA, respectively, was incubated with HiPerFect from Qiagen in RPMI 1640 medium free of FBS for 20 min at room temperature and then added to freshly seeded cells.

### *POLD1* knockout with CRISPR/Cas9

The CRISPR *POLD1* Knockout Kit was purchased from OriGene (Rockville, MD, USA), containing two pCas9 plasmids (targeted sequence of gRNA1 TGCCCCCAAAGCGGGCCCGT and gRNA2 GGGATGATGATGATGCACCT, respectively), one pCas9 plasmid with a scrambled sequence as ctrl and a repair template with a left homology arm (LHA) and right homology arm (RHA) of *POLD1* together with a GFP-Puromycin functional cassette (Figure S4). DLD-1 cells were reverse co-transfected using 1 μg of the repair template together with 1 μg of one of the three pCas9-Guide vectors. 72 h later the transfection complex containing medium was replaced with puromycin containing medium for selection of stably transfected cells. Selected cells were allowed to grow in single-cell colonies and genotyped, using the primers #1–#4 (Table S1). Heterozygous clones show two PCR products, one representing the KO allele (approx. 1000 bp) and the other one representing the intact wild type (WT) allele (1656 bp). To engineer homozygous KO clones, heterozygous clones were first transfected with a Cre vector to remove the puromycin resistance gene. Single cell colonies with removed puromycin resistance gene were verified via PCR using the primers #5 and #6. Single-cell colonies with no puromycin resistance gene were then repeatedly transfected as described, applying three independent targeting rounds with at least 80 genotyped single-cell colonies during each targeting round.

### PCR and sequencing

DNA and RNA were isolated using the Mini Kits QIAamp and RNeasy from QIAGEN, respectively. RNA to complementary DNA transcription was performed using the Omniscript RT Kit from Qiagen. For the PCR reactions Promega’s GoTaq G2 DNA Polymerase was used (Madison, WI, USA). Primers are listed in Table S1 and PCR conditions are available on request. Sequencing was performed by GATC Biotech AG (Constance, Germany), samples were prepared as asked by the provider. Primers used for sequencing are listed in Table S2.

### Immunoblotting

Immunoblotting was performed as described previously^39^. In short, cells were lysed and protein extracts were boiled and loaded on 10% polyacrylamide gels. After electrophoretic separation, the proteins were transferred to PVDF membranes, which were blocked with 5% milk powder in Tris-buffered saline + 0.1% Tween 20 (TBS-T) for 1 h. Incubation of primary antibody in TBS-T was performed at 4 °C overnight. Membranes were then washed and stained with secondary antibody. Chemiluminescence was elicited using Western Lightning Ultra from PerkinElmer (Waltham, MA, USA) or Clarity Western ECL Substrate from Bio-Rad Laboratories (Hercules, CA, USA), respectively, according to the manufacturers’ instructions. The following primary antibodies were used: anti-caspase 3, anti-cleaved caspase 3 (Asp175), anti-PARP, and anti-pCHK1(Ser345) (133D3) from Cell Signaling (Cambridge, UK), anti-CHK1 (G-4) and anti-POLD1 (A9) from Santa Cruz Biotechnology (Dallas, TX, USA), and peroxidase-conjugated anti-β-Actin (AC-15) from Sigma-Aldrich (Hamburg, Germany). HRP-conjugated anti-rabbit, anti-goat and anti-mouse antibodies from Santa Cruz Biotechnology were used as secondary antibodies.

### DNA fiber assay

Exponentially growing cells were pulse labeled with 25 μM chlorodeoxyuridine (CldU) followed by 250 μM iododeoxyuridine (IdU), both from Sigma-Aldrich (Hamburg, Germany), for 30 min each. For analysis of fork stability cells were treated with 2 mM HU for 4 h or irradiated with 6 Gy between both labels. Labeled cells were harvested and DNA fiber spreads prepared and stained as described^45–47^. Fibers were examined using an Axioplan 2 fluorescence microscope from Carl Zeiss (Jena, Germany). CldU and IdU tracks were measured using ImageJ and micrometer values were converted into kilobases. At least 100 forks were analyzed. Different classes of labeled tracks were classified: red-green (ongoing replication), red (stalled forks) and green (2nd pulse origin). Labeled tracks were counted using ImageJ and at least three independent experiments were performed.

### Analysis of cell cycle and apoptosis

Cells were seeded and allowed to adhere overnight before treated with 1 µM AZD6738. After 96 h of treatment, cells were collected, washed, and stained with propidium iodide (PI) (0.1% sodium citrate, 0.1% Triton X-100, and 50 µg/ml PI) as described previously^48^. For analysis of apoptosis, cells were collected at various time points after treatment with 1 µM AZD6738, washed with cold Hank’s Balanced Salt Solution, and stained for 20 min in the dark at room temperature with 25 µl/ml of a FITC-conjugated annexin V-antibody from Biolegend (San Diego, CA, USA). Directly before flow cytometric measurement, 10 µg/ml PI was added to the staining solution. Cell cycle distribution and apoptotic cells were quantified by using the BD FACSCanto II from BD Biosciences (San Jose, CA) and the FlowJo v10 software from FlowJo, LLC (Ashland, OR). At least 20,000 gated events per sample were analyzed.

### Cell proliferation assays

Cell proliferation assays were performed over a broad range of concentrations covering 100% to 0% cell survival. 1500–1800 cells of the DLD-1 *POLD1*^+*/*+^ parental and ctrl clones as well as of the *POLD1*^*R689W/-*^ and *POLD1*^+*/-*^ clones were plated in 96 well plates and allowed to adhere overnight, before being treated with various drugs at multiple concentrations for 120 h. Following incubation, the cells were washed, lysed in 100 μl H_2_O and 0.2% SYBRGreen (Lonza, Cologne, Germany) was added. Fluorescence was measured using a Victor3 V plate reader (PerkinElmer, Waltham, MA, USA) and growth inhibition was calculated as compared to the untreated control samples.

### Xenograft model

Xenograft tumors were induced in 6 week old female nude NMRI-*Foxn1*^*nu/nu*^ mice from Charles River Laboratories (Wilmington, MA, USA) by subcutaneously injecting 10^6^ *POLD1*^+*/*+^ or *POLD1*^*R689W/-*^ DLD-1 cells, respectively, suspended in phosphate buffered saline. Two weeks after injection, mice were randomly divided into two groups for each cell clone and the ATR inhibitor AZD6738 at a dose of 50 mg/kg or the vehicle solution alone were administered by gavage once daily for five consecutive days followed by two days of no administration. This application schedule was conducted for four weeks (Fig. 6A). Solid tumors were measured twice a week with calipers and the calculated tumor volume ($V=x\cdot {y}^{2}\cdot 0.5 \text{with }x=\text{longest diameter, }y=\text{shortest diameter}$) on day one of the application schedule was set as reference to calculate the percentage change of tumor volume. Additionally, mice were weighed before every inhibitor/vehicle application. AZD6738 was diluted in 100% dimethyl sulfoxide (DMSO) and the vehicle solutions consisted of 10% DMSO (with or without AZD6738), 40% 1,3-propanediol and 50% H_2_O.

All animal experiments were performed according to the guidelines of the German law for animal life protection and approved by the commission for animal protection of the veterinary office of the regional commission of Gießen with the file numbers G44/2017 and G89/2018.

### Statistical analysis

All statistical analyses were performed using Prism 5 from GraphPad Software Inc. (La Jolla, CA, USA). Surviving fractions of the proliferation assays were calculated by curve fitting with nonlinear regression. Using Grubbs’ test statistically significant outliers were determined and not included. Data are presented as mean ± SEM, and a one-tailed or two-tailed, unpaired Student’s t-test, or a two-way ANOVA with Bonferroni post-test were used for statistical interpretation. P-values of p < 0.05 (*), p < 0.01 (**) or p < 0.001 (***) were considered statistically significant.

## Supplementary information

Supplementary Information.

## Data Availability

The datasets used and/or analysed during the current study are available from the corresponding author on reasonable request.
