# Supplementary material for: The POLD1R689W variant increases the sensitivity of colorectal cancer cells to ATR and CHK1 inhibitors
Source: Sci Rep. 2020 Nov 3;10:18924. doi: 10.1038/s41598-020-76033-1 (PMC7641191; doi:10.1038/s41598-020-76033-1)
Supplement: Supplementary file 1 — Supplementary Information. [file 41598_2020_76033_MOESM1_ESM.pdf]

The *POLD1*^{R689W} variant increases the sensitivity of colorectal cancer cells to ATR and CHK1 inhibitors

Albert Job[1], Marina Tatura[1], Cora Schäfer[1], Veronika Lutz[1], Hanna Schneider[1], Brigitte Lankat-Buttgereit[1], Alexandra Zielinski[2], Kerstin Borgmann[2], Christian Bauer[1], Thomas M. Gress[1], Malte Buchholz[1], and Eike Gallmeier[1,*]

[1]Department of Gastroenterology, Endocrinology, Metabolism, and Infectiology, University Hospital of Marburg, Philipps-University Marburg, Marburg, Germany
[2]Lab of Radiobiology & Experimental Radiooncology, University Medical Center Hamburg-Eppendorf, Hamburg, Germany
[*]eike.gallmeier@med.uni-marburg.de

Supplementary Information

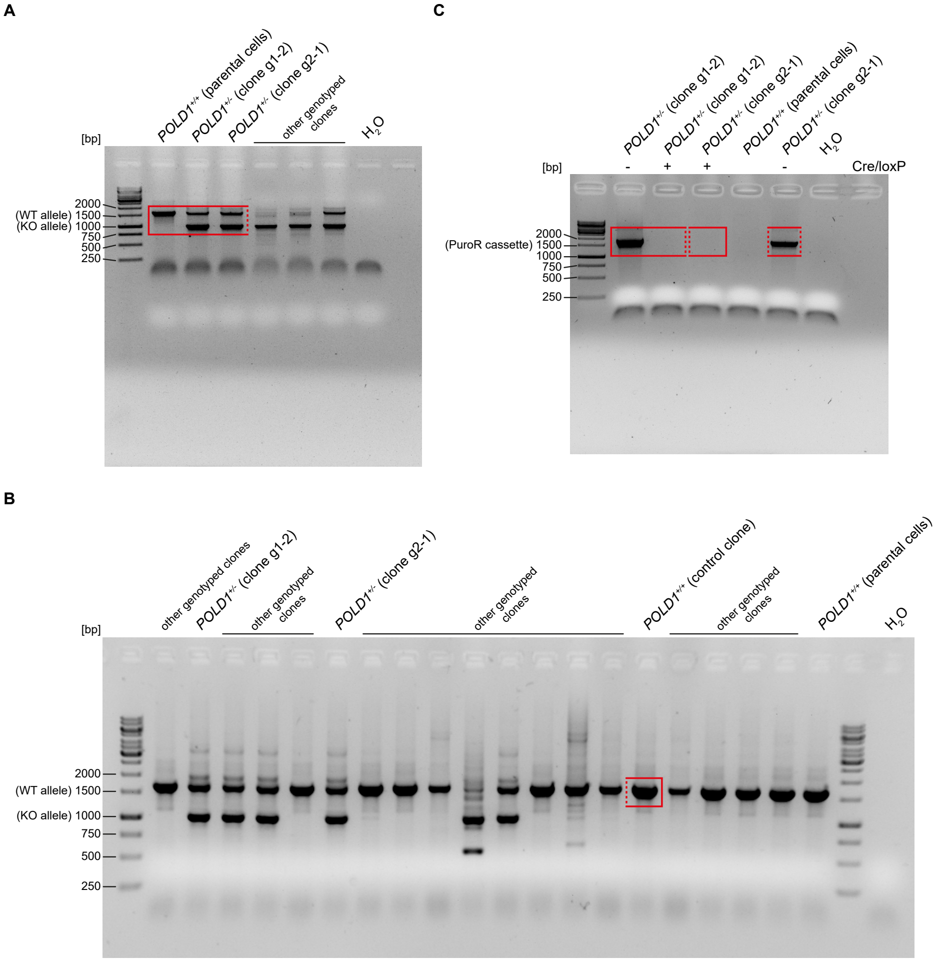

Figure S1. (**A+B**) Original gels of the PCR detecting the *POLD1*-KO and -WT alleles in the *POLD1*$^{+/+}$ parental and control clones as well as in the *POLD1*$^{+/-}$ clones g1-2 and g2-1. Data which were cropped and depicted in Figure 1B are framed red. (**C**) Original gels of the PCR detecting the Cre/loxP-mediated excision of the puromycin resistance cassette. Data which were cropped and depicted in Figure 1C are framed red.

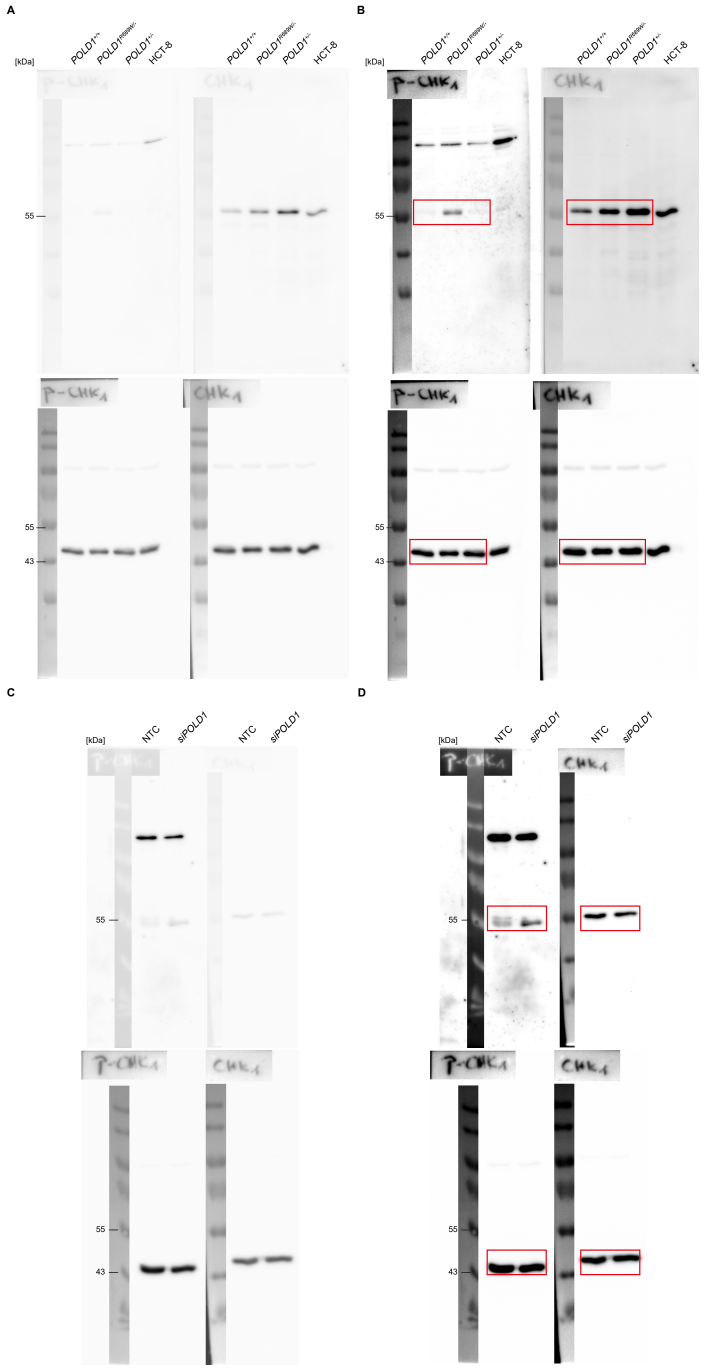

Figure S2. (**A, upper panel**) Original immunoblots and (**B, upper panel**) immunoblots after contrast modification of constitutive protein levels of pCHK1 and CHK1 in *POLD1*$^{+/+}$, *POLD1*$^{R689W/-}$ and *POLD1*$^{+/-}$ cells. (**A+B, lower panels**) Original immunoblots and immunoblots after contrast modification, respectively, of β-Actin serving as loading control. (**C, upper panel**) Original immunoblots and (**D, upper panel**) immunoblots after contrast modification of protein levels of pCHK1 and CHK1 120 hours after *siPOLD1* transfection in *POLD1*$^{+/+}$ cells. (**C+D, lower panels**) Original immunoblots and immunoblots after contrast modification, respectively, of β-Actin serving as loading control. Data which were cropped and depicted in Figure 3A are framed red.

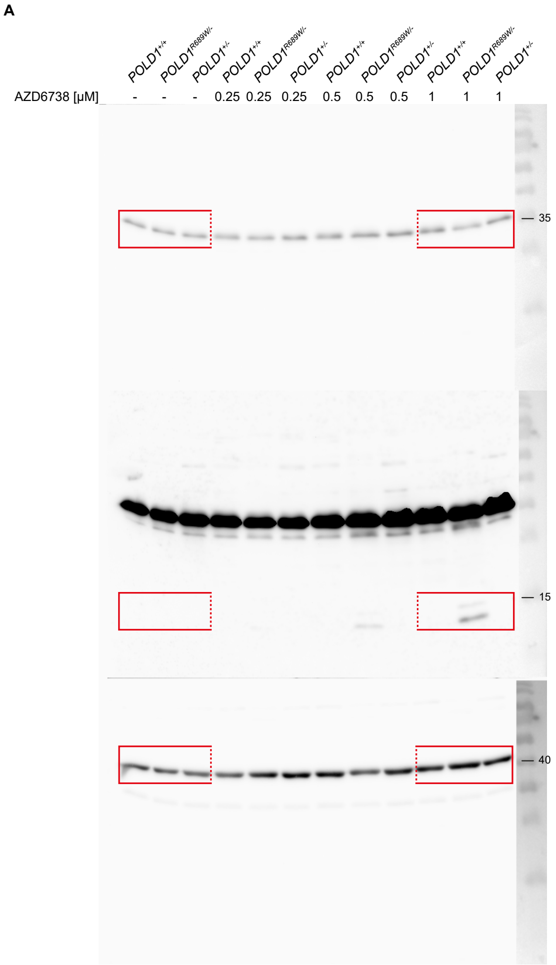

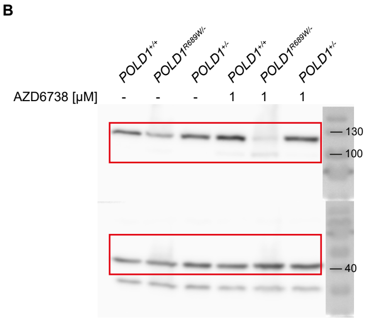

Figure S3. Original immunoblots of protein levels of (**A, upper part**) caspase 3, (**A, middle part**) cleaved caspase 3 as well as (**B, upper part**) PARP and cleaved PARP in *POLD1*$^{+/+}$, *POLD1*$^{R689W/-}$ and *POLD1*$^{+/-}$ cells 72 hours after treatment with AZD6738. (**A+B, lower parts**) Original immunoblots of β-Actin serving as loading control. Data which were cropped and depicted in Figure 4C are framed red.

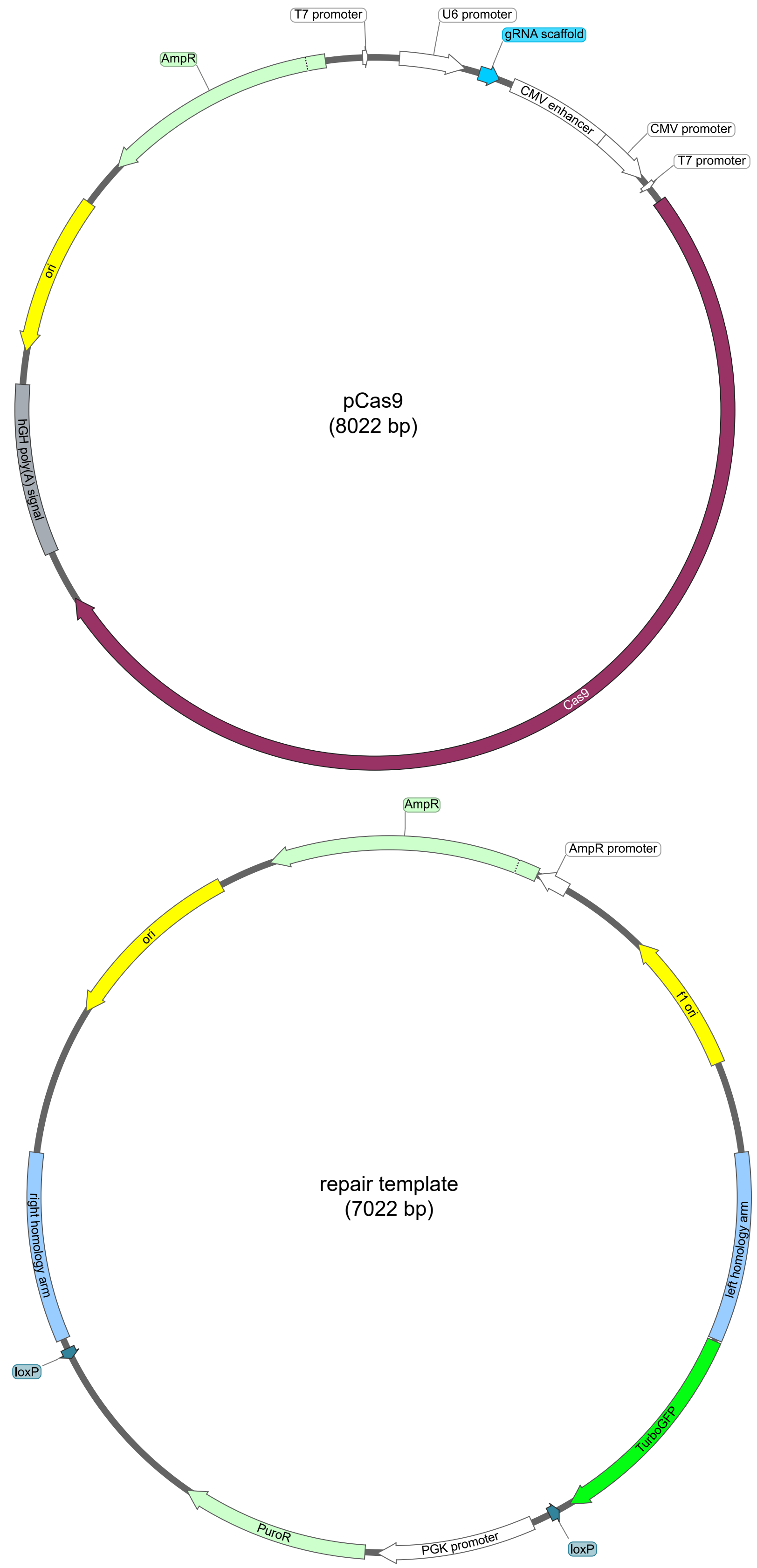

Figure S4. Maps of the pCas9 and repair template plasmids.

Table S1. **Primer used in PCR**

Primer	Orientation	Localization	Sequence (5' - 3')
# 1	sense	POLD1 NCS[a], upstream LHA	GTGAGAGAGCACACACACGAC
# 2	antisense	repair template, GFP	TAGGTGCCGAAGTGGTAGAAGC
# 3	sense	repair template, spacer region	TCTCTTGATTCCCACTTTGTGGT
# 4	antisense	POLD1 NCS, downstream RHA	CAGATCAACGCTCCAAGCAC
# 5	sense	POLD1, LHA	GAGGTGTCTCCGGTCAGAAC
# 6	antisense	repair template, puromycin resistance	GAGGCCTTCCATCTGTTGCT
# 7	sense	POLD1 NCS, upstream exon 13	CCCAGACCCTGACGACTTGG
# 8	antisense	POLD1 NCS, downstream exon 13	TGGGAGTGGGGAGAAAAAGTG
# 9	sense	POLD1 NCS, upstream exon 17	TGCGTGAATTAGCACAAGGC
# 10	antisense	POLD1 NCS, downstream exon 17	GGACCAATTGCTCAAGCCAC
# 11	sense	POLD1 NCS, upstream exon 18	TCCGCATGATTCTCTCCCCG
# 12	antisense	POLD1 NCS, downstream exon 18	GTGGCTAATGCCAACGGGAC
# 13	sense	POLD1, exon 2a	GGGCCTCTGGGATGATGATG
# 14	antisense	POLD1, exon 21	CTGGGAGATATCGATGCGGT
# 15	sense	POLD1, exon 10	GTATCATGGACCCCGACGTG
# 16	antisense	POLD1, exon 14/15b	CGTAGTACCCTTTGAGGGGC
# 17	sense	POLD1, exon 15b	CTGTGTTACACCACGCTCCT
# 18	antisense	POLD1, exon 20	GTGAGGCAGTGACCAGGTTG

[a] NCS: non coding sequence

Table S2. **Primer used for sequencing**

Primer	Localization	Sequence (5' - 3')
# S1	upstream exon 2a, NCS[a]	TCAGAACCTCCACCAAG
# S2	upstream exon 13, NCS	ACTTCCTTCTCCTGCTC
# S3	upstream exon 17, NCS	TGTGCAGTGCACAGTAC
# S4	upstream exon 18, NCS	GTTCGGACGTCAGATGATC
# S5	upstream exon 13	CTCCTACACGCTCAATG
# S6	upstream exon 17	AGATCCTGGAGAACCTG

[a] NCS: non coding sequence
